# Addressing vaccine hesitancy requires an ethically consistent health strategy

**DOI:** 10.1186/s12910-018-0322-1

**Published:** 2018-10-24

**Authors:** Laura Williamson, Hannah Glaab

**Affiliations:** Biobehavioral Health Department, Pennsylvania State University, University Park, USA

**Keywords:** Vaccination, Public health, Public policy, Hesitancy, Engagement, Ethics, Deliberation

## Abstract

**Background:**

Vaccine hesitancy is a growing threat to public health. The reasons are complex but linked inextricably to a lack of trust in vaccines, expertise and traditional sources of authority. Efforts to increase immunization uptake in children in many countries that have seen a fall in vaccination rates are two-fold: addressing hesitancy by improving healthcare professional-parent exchange and information provision in the clinic; and, secondly, public health strategies that can override parental concerns and values with coercive measures such as mandatory and presumptive vaccination.

**Main text:**

It is argued that such conflicting, parallel approaches seriously risk undermining trust that is crucial for sustaining herd immunity. Although public health strategies can be ethically justified in limiting freedoms, a parent-centered approach seldom acknowledges how it is impacted by contemporaneous coercive measures. In addition, the clinical encounter is not well suited to helping parents consider the public dimensions of vaccination, despite these being important for trust formation and informed decision-making. Efforts to address vaccine hesitancy require more consistent engagement of parental and citizen views. Along with evidence-based information, debates need to be informed by ethical support that equips parents and professionals to respond to the private and public dimensions of vaccination in a more even-handed, transparent manner.

**Conclusion:**

Efforts to address vaccine hesitancy need to avoid simple reliance on either parental values or coercive public policies. To do this effectively requires increasing citizen engagement on vaccination to help inform a parent-centered approach and legitimize public policy measures. In addition, cultivating a more ethically consistent strategy means moving beyond the current silos of health ethics - clinical and public health ethics.

## Background

### Disease outbreaks and vaccination uptake

Childhood vaccination is a critical and highly effective component of maintaining public health. Rates of immunization have remained generally high in developed countries, but persistent clustering of low levels of uptake and associated disease outbreaks are increasingly problematic [1]. For example, the measles outbreak (2014–15) linked to Disneyland in California, United States of America (U.S.) was found by a modelling analysis of the infected population to have resulted from vaccination rates as low as 50% and no higher than 86% [2]. Similarly, the World Health Organization (WHO) has recently expressed concern about measles outbreaks across Europe associated with low vaccination uptake - including 41,000 cases in children and adults across the region in the first 6 months of 2018. Finding ways to secure high levels of childhood vaccination - at least the 95% recommended by the WHO - to sustain herd immunity is a public health priority [3].

### Vaccine anxieties and hesitancy

An obstacle to the uptake of childhood vaccination is long-standing anxieties towards it. Citizens’ concerns relate to a wide variety of issues, including the: perceived safety of vaccination; liberty restricting nature of legislation that seeks to mandate vaccination coverage; motives of industry in developing new vaccines; expansion of already sizable childhood vaccine schedules; and perceptions of professional expertise and authority [4]. Such worries are now recognized as leading to growing levels of ‘vaccine hesitancy’. That is, parental assessments of immunization reflect a degree of psychological ‘indecision’ over whether to proceed with vaccination [5]. Parents with little information on vaccination as well as those who are informed, but seek to supplement their knowledge, can be vaccine hesitant [6, 7]. Hesitancy leads to a variety of behaviors including: under vaccination of children; delaying vaccination uptake; and rejecting some vaccines, while accepting others [8]. Concerns have been expressed that the term ‘vaccine hesitancy’ has become ambiguous, or a ‘catchall category’ that creates obstacles for efforts to address it [6]. Thus, it necessary to emphasize that, following others, our examination of vaccine hesitancy does not include parents facing structural obstacles to accessing vaccinations, nor those parents with an established non-vaccination position [5, 6].

We focus our discussion on vaccine hesitant parents for two interrelated reasons. Firstly, it has been argued that the most efficient way to address low uptake is to persuade hesitant parents, or ‘fence-sitters’ to vaccinate [9]. Secondly, there are underexamined ethical issues regarding how the concerns of hesitant parents are addressed across clinical and public policy contexts – these have ramifications for trust. This is important because vaccine hesitancy is influenced by the level of trust people have in their healthcare provider, or wider social systems [6, 8, 10]. To accept vaccines readily, people need confidence that, in the face of uncertainty and risk, professionals have their best interests at heart [11]. Without this foundation, people can be ‘suspicious of the motives and practices’ of those they engage with [11]. Indeed, trust or lack thereof, have been reported as one of the main ‘determinants of mothers’ decisions about vaccination’ [12]. When people feel listened to, understood and communicated with openly their trust in Healthcare professionals (HCPs) is enhanced [13].

### Promoting trust through parent-centered care

Efforts to cultivate trust in vaccination primarily draw on the concept of person, or in the case of childhood vaccination, parent-centered care (PCC). Though we will argue below that this approach is undermined by public health measures. A PCC approach requires professionals to be ‘respectful of and responsive to individual patient preferences, needs, and values’ and aims to ensure ‘patient values guide all clinical decisions’ [14]. HCPs and service users work together to help ensure health decisions are based on the best available evidence and the values of those seeking health services [15].

In the context of vaccination parents report they want HCPs to listen to their vaccination concerns and answer questions they have [16]; and to use a consultation style that cultivates ‘open, non-confrontational dialogue’ [17]. Two-way exchange between HCPs and parents is fundamental to supporting informed decision-making and allows vaccination consultations to exploit the crucial role that HCPs have in determining whether parents choose to immunize their children [17, 18]. This is partly because HCPs serve as a direct point of contact for parents making such decisions. In addition, HCPs are consistently shown to be ‘a trusted source of vaccine information’ [19].

An important requirement in clinical contexts is informed consent which traditionally requires five elements: competent decision-makers, adequate disclosure of information relating to risks and benefits, understanding of the information provided, voluntariness and the actual consent for an intervention [20]. Informed consent has been presented as a tool for helping to support parental engagement and trust building in the vaccination encounter [16]. Indeed, there is a close relationship between promoting understanding as the basis for cultivating trust and gaining informed consent which is best achieved by ‘establishing a climate that encourages’ people to ask questions, rather than relying on the one-way delivery of large amounts of information [21]. Despite the importance of informed consent, we suggest that within vaccination consultations freely given consent can be impeded, often without acknowledgment, by parallel public health measures. This can place a strain on HCPs given their commitment to follow consent requirements.

### Using coercion or ‘nudging’ to promote vaccination

Efforts to address vaccine anxieties through exchanges between parents and HCPs run in tandem with approaches that coerce or ‘nudge’ parents to vaccinate their children. In the context of vaccination, coercive measures take a variety of forms internationally including: vaccine mandates; the recent ‘No jab, no pay’ policy in Australia, that seeks to use financial pressure to promote vaccination [22]; and HCPs barring non-vaccinated children from their surgery in the U.S. [23]. Such coercive threats to get people to vaccinate, are intended to be irresistible. However, as Faden and Beauchamp argue, coercive measures are subjective because people have varying capacities to resist them [24]. For example, some families are able to home school their children, or pay for private schools, while for others these options are out of reach. Similarly, efforts to influence or ‘nudge’ parents by using means such as managing the way information is presented, or not presented, to them also bypasses the meaningful elucidation of parental concerns promised by PCC.

## Main text

In what follows we begin by outlining concerns that competing vaccination strategies ethically conflict, before turning to examine related issues in more detail: the variable experience of PCC; the increasing use of mandatory vaccination; and, proposals for a ‘presumptive’ approach in vaccination consultations. In the final main section of the paper, we advance to examine the need to find ways to rebalance ethical commitments at the heart of efforts to address vaccine hesitancy and increase immunization uptake. In this respect, we argue that if trust in vaccination is to be built and sustained in the longer term, it is essential to formulate an integrated, consistent ethical approach to vaccination policy and practice.

### Inconsistent, conflicting vaccination strategies

Despite the prominence of arguments for a PCC approach in vaccination literature, its variable delivery in clinical environments and tension with parallel public health strategies that bypass engaging parents, create a confused ethical climate. Parents are led to believe that their views are ethically and practically important. However, they can then experience their wishes and concerns - or witness those of others - being overridden by efforts to pressurize them to vaccinate. This situation risks being counterproductive for efforts to build trust in vaccination and the health systems that provide them.

#### Variable experience of vaccination consultations

Despite the espousal of a need for parent-centered consultations to address the fundamental drivers of vaccine hesitancy, a participatory approach is not always experienced. In this respect, Brown et al. found that parents report ‘feeling condescended to’ and experience ‘unequal power relations prohibiting free discussion’ and, as a result, ‘dissatisfaction’ with vaccination consultations [25]. Significantly, it is not only parents who can report uneasiness with vaccination consultations, HCPs can find it hard to respond to patients (parents) when they disagree about evidence-based recommendations [26]. In addition, HCPs express concern that encouraging parental questioning will ‘open up a can of worms’ and lead to vaccination refusal [27]. This is despite research that shows values clarification with parents does not necessarily result in non-vaccination and can help to minimize post-vaccination anxieties (or ‘decision-regret’) [28]. It is also troubling that Kennedy et al. portray questioning by parents as one of the ‘signs of reduced confidence’ in vaccination [18], rather than reasonable parent-centered inquiry.

The inconsistency that can characterize parental-HCPs exchange around vaccination risks impeding efforts to build trust. This is partly because not directly eliciting questions to address parental concerns can miss opportunities to allay anxieties. In addition, lack of dialogue in the clinic – whether due to anxieties or logistical pressures - itself risks promoting distrust as it breaches commitments and expectations surrounding participatory exchange in health contexts. Such commitments have increasingly led to people expressing a desire to have the option of taking part in decisions that impact on their health [29, 30]. A failure to meet such expectations risks promoting dissatisfaction with service provision and impeding trust promotion in, and future engagement with, health services. However, the utilization of PCC to promote trust in vaccination encounters a greater challenge when top-down public health measures, that aim to pressurize parents to vaccinate their children, are employed. More specifically, the increasing use of vaccination mandates and proposals for ‘presumptive’ vaccination seek to impose immunization without addressing parental concerns or respecting their values.

#### Mandatory vaccination: Undermining parent-centered care and consent

In the U.S. all states mandate vaccination for school entry, though the precise requirements differ [31]. States have traditionally offered different exemptions to mandates, although recent disease outbreaks have prompted a growing number to seek to withdraw such exemptions [32]. In Europe, France and Italy have recently extended their use of mandatory vaccination [33, 34]. While there has been discussion about mandating vaccination in the United Kingdom (U.K.), the option has been rejected [35]. Mandates are implemented by way of penalties for non-compliance. As a result, there is pressure on parents to vaccinate their child or accept a penalty such as having them barred from school. Thus, when parents attend for a vaccine consultation their choice regarding immunization can be pressurized within mandated systems and not a free, uncoerced decision as informed consent processes require.

It is well established that public health ethics and law can justify the infringement of liberties to protect public health if the restrictions are proportionate and necessary [36, 37]. Thus, coercive vaccination policies may be justifiable depending on the circumstances, such as a severe disease outbreak. It has been argued that such mandates are ethically justifiable to protect individual children and the common good [38]. However, there are concerns that overriding informed consent requirements can lead to ‘counterproductive resistance’ towards public health strategies [39]. Yet as Gostin suggests, the right to informed consent does not allow one to ‘override the rights of others to live safely in their communities’ [40]. The implication being that transparent restrictions can, when circumstances demand, be placed on informed consent to protect public health with measures such as vaccine mandates. But the ethical soundness of this claim is impacted by factors such as the availability of alternatives and the potential for coercion itself to undermine these options without sufficient consideration or acknowledgment. More specifically, without work to ease the tension between PCC and public health approaches to vaccine hesitancy that run in tandem, conflicting ethical approaches (coercion vs parental permission) can result in vaccination strategies that risk being counterproductive. This inconsistency threatens to undermine efforts to build confidence in vaccination systems in the longer term. As Widdus and Larson have recently highlighted, coercive public policy needs to consider the ‘law of unintended consequences’ [41].

Significantly, that PCC strategies are impacted by, but do not acknowledge the impact of parallel coercive approaches on their ethical commitments can have implications beyond countries with mandatory policies. This is because discussion of health strategies contrary to the prioritizing of PCC and consent (parental permission) in the public forum has the potential to impact more widely. This is apparent with work on ‘presumptive’ vaccination to which we now turn.

#### Presumptive vaccination

Debates on ‘presumptive’ vaccination consultations also reveal a deep conflict over the importance afforded to parental anxieties and values [19, 42]. ‘Presumptive’ consultations involve HCPs trying to secure vaccination uptake by making a statement such as ‘Well, we have to do some shots’, rather than asking parents about their preferred course of action [19]. Opel et al. found that when used by HCPs, a ‘presumptive’ consultation format results in higher rates of vaccination uptake than a participatory approach [19]. Although Opel et al. were aware that their findings challenged fundamental features of participatory clinical practice, they suggested such approaches may need to be ‘reconsidered’ if presumptive vaccination increased uptake [19]. In response, Leask et al. argued that the research misrepresented the requirements of participatory decision-making and so underestimated its value; and could lead to less vaccination because of its negative impact on trust and promotion of ‘acquiescence’ [42].

At of heart of concerns about this practice is that parents are ‘nudged’ to vaccinate their children without their knowledge, though in ways they could resist – by refusing vaccination. More specifically, ‘nudging’ seeks to make certain choices the ‘default’ option by changing the ‘architecture’ in which choices are made [43]. Faden and Beauchamp argue that efforts to control peoples’ decisions by intentionally managing the information they are given can undermine their ‘understanding’ and so impair autonomy [24]. Similarly, Ploug and Holm in their examination of ‘nudging’ in the clinic contend information control is ‘incompatible with a proper protection of personal autonomy through informed consent’ [44]. However, it has been contended that concerns about ‘nudging’, or more broadly, ‘nonargumentative influence’, need to be unpacked carefully to assess their ethical standing. In this respect, a more precise consideration suggests that not all ‘nonargumentative influence’ impedes autonomy [45]; indeed, in some cases it can ‘aid values clarification’ [46]. More specifically, to determine whether actions like information control are morally acceptable requires assessing: the awareness of and attitude of the influenced towards the influence; whether a deciders options are blocked; if the relationship between influencer and influenced would be damaged; and whether the influence would be expected in the context of the relationship in question [45]. While such considerations may make information control acceptable for some issues, we will now suggest that, for a number of reasons, this does not apply to vaccine hesitancy.

Firstly, the diversity of vaccine hesitancy means that while some parents may not object to control of information to intentionally direct their decisions, or not consider their decisions blocked, other parents will object to both strongly. Awareness of the intended influence is also likely to vary based on parents’ knowledge of the commitments of PCC and perhaps awareness of research on ‘presumptive’ vaccination, which we discuss shortly. Similarly, hesitant parents will have differing expectations of the level of transparency they demand from their HCPs. For this reason, it is not possible to generalize the use of presumptive vaccination without creating ethical concerns amongst some, perhaps many, hesitant parents. Secondly, a key feature of hesitancy is lack of trust and a corresponding imperative to build confidence [6]. This suggests there is a need for vaccination systems to be seen to respect widely accepted clinical standards and promised commitments to PCC. Outside of an emergency situation, normalizing policies that may not be thought adhere to standards of transparency, respecting the wishes of parents (patients) risks undermining trust and damaging relationships – unless citizen acceptance of such policies has been obtained. Thus, despite claims that ‘nudging’ can help in values clarification [46], in the context of vaccination, this is unlikely to be feasible in circumstances where doubts have not been acknowledged and resolved.

As alluded to above, confidence in vaccination systems is also potentially impacted by media articles relating to the publication of the study by Opel et al. that included headlines such as: ‘To get parents to vaccinate their kids, don’t ask. Just tell’ [47]. In the volatile context of declining vaccination this message could, internationally, promote doubts in the minds of parents about whether vaccination will be imposed if they attend for a consultation, and so impede their engagement with health services. That such concerns threaten to undermine trust and exacerbate vaccine hesitancy is unfortunate. This is not least because in a later study linking parental satisfaction to a participatory consultation style, Opel et al. acknowledge, despite the ability of presumptive vaccination to increase uptake ‘… it may be that participatory initiation formats are a better match for the development of an open, trusting relationship that parents seek’ [48]. This, like the other ethical tensions we have identified, highlights the importance of thinking strategically across clinical and policy contexts about the long-term development of vaccination policy and practice to avoid different features becoming self-defeating.

### Rebalancing ethical commitments in vaccination strategies

In addition to concerns that PCC is undermined by inconsistent delivery and conflicting policy approaches, there are also fundamental questions regarding whether its focus on narrow, private parental interests is sufficient to attend to the wider public dimensions of vaccination. This concern is evident in the context of debates on herd immunity and is now discussed.

#### Herd immunity: Supplementing parent-centred care

Public health concepts like herd immunity can struggle to gain attention in the parent-HCP encounter. This is because parents have been found to show little interest in herd immunity and tend to focus solely on their private interests relating to their child [49, 50]. Lack of parental interest in herd immunity has led to claims that ‘invoking herd immunity does not increase pediatric vaccination rates’ [51]. However, knowledge about herd immunity is critical for informed vaccination decisions and failure to engage parents on such issues does a disservice to their children and public health. Thus, it is important that PCC avoids uncritical deference to potentially uninformed parental choices. Indeed, work on PCC itself highlights the need to address parents (or patients) within their wider social contexts [52, 53].

It is necessary to moderate the prominence of parental choice by highlighting that informed decisions are formed relationally. To support such commitments, a ‘relational’ concept of autonomy should become standard within consultations [54, 55]. That is, an account that emphasizes informed, autonomous decisions are made, not in isolation, but with the support of others. Integrating such support would highlight that interconnection is fundamental to informed decision-making, and not necessarily an obstacle to it. This perspective offers a way to help ensure professional expertise is valued in clinical and public health contexts; and, as a result, is able provide an opportunity to expand the attention afforded to herd immunity. In this, our primary focus is to help support and inform the decisions of undecided, vaccine hesitant parents. However, embedding relational autonomy at the heart of immunization debates could also help to create an ethical climate in which non-vaccinating behaviours like ‘free-riding’ are less acceptable.

#### Public deliberation on vaccination

Vaccine hesitancy is also impacted by issues requiring wider public debate including lack of trust in government or pharmaceutical companies, and policies that alter the balance between individual choice and public interest. However, it is not logistically feasible for all these issues to be addressed in clinical vaccination consultations. Importantly, if parents are only involved in vaccination decisions as private individuals in the clinic, the public dimensions of immunization – including values-based challenges - risk being overlooked in their reasoning. This is despite the crucial role public issues have in informed decision-making. We now turn to examine how increasing public deliberation on vaccination has an important role to play in cultivating ethically consistent vaccination strategies, that are better equipped to promote and sustain trust in the longer term.

Vaccination policy is marked by a tendency to default to traditional, top-down public health approaches that prioritize determining when it is appropriate to place limitations on parental permission. We have argued that, in its present form, this ethically and practically undermines commitments to addressing vaccine hesitancy by utilizing a PCC approach. It also disregards a wider ‘deliberative turn’ in health policy which seeks to deliver on the WHO’s 1978 claim that people ‘have a right and duty’ to participate ‘collectively’, as well as individually, in debates that impact their health [56, 57]. Alternative approaches to supporting public health by actively involving citizens in policy formation are available [53]. Indeed, recently public consultation has played an important role in the extension of mandatory vaccination in France [58]. Yet, as the actual or proposed attempts to tighten mandates without civic debate in the U.S. show [59], involving citizens in vaccination policy development is still uncommon. It has been acknowledged that contemporary vaccination debates afford little attention to citizen participation [60–62]; and proposed that civic deliberation could be utilized in the context of vaccination [61, 62], though the suggestion remains underdeveloped. In what follows we support the claim that civic deliberation could be usefully employed as part of an improved response to vaccine hesitancy. More specifically, we contend that the use of civic deliberation is critical for the development of ethically consistent responses to vaccine hesitancy that are able to win and sustain trust in the longer term.

Public deliberation aims to develop inclusive, respectful, reasoned policy positions on contentious issues in ways that all participants can understand, if not necessarily agree with, based on information they find accessible [63]. In this way, deliberation systematically examines tensions and disagreements by drawing on ‘persuasion rather than coercion, manipulation, or deception’ [56]. Thus, the approach could help to develop policies to address vaccine hesitancy that address parental concerns. More specifically, deliberative events could require parents to examine tensions between the private and public dimensions of vaccination which cannot be covered in clinical encounters. The precise focus of and methods employed by such civic initiatives and can be determined by local need and resources – whether school district, health care facility or wider geographic area. However, issues suited to deliberative debate could include the expansion of vaccine schedules, and whether the extension of public health strategies - like mandatory and presumptive vaccination - are publicly acceptable and conducive to sustaining trust. Beyond the substantial literature base that exists on deliberative theory that could help inform debates on vaccine hesitancy, there is also much useful material on the application of deliberation within and beyond the health field [64–66].

In the context of vaccine hesitancy public deliberation would facilitate parents to work through doubts about vaccination and tensions between individual choices and public health using reasoned debate. This approach differs from other civic engagement strategies because it goes beyond citizens merely sharing their opinions. In addition to information provision and facilitated civic debate, deliberation has an important role to play in promoting trust given its capacity to ‘promote the legitimacy’ of public decisions, despite underlying tensions in the community [63]. That is, once a civic group reaches a decision on a contentious issue, the informed, transparent, reasoned way the conclusion is reached can help those who do not agree with the outcome in accepting it. In the context of vaccination, while disagreement may well remain after deliberation, the approach alleviates concerns immunization systems lack transparency and are untrustworthy.

Associated with the aim of deliberation to win legitimacy amongst diverse perspectives are concerns that debates can become ‘standoffs’ between opposing sides of an issue [63, 67]. In this respect, social media exchanges on vaccination evidence the potential for heated exchanges. However, the ground rules of deliberation and skilled facilitation aim to provide the best possible environment for addressing disagreements. In the context of vaccination, trust in deliberative processes is supported by the fact that the results of such exercises could differ significantly. For example, deliberation offers a way for vaccine hesitant parents to subject strategies with which they do not agree – perhaps the routine overriding of parental permission by vaccine mandates – to critical scrutiny. This could help avoid the perceived need for coercive strategies to get people to vaccinate or win public approval for such approaches, or measures like incentivization. It should be noted that public health would still retain police powers for emergency situations.

Public deliberation is necessary for improved responses to hesitancy, not only because to can help people debate difficult issues and legitimize contentious policies, it also requires parents to participate as citizens. This is important because parents are rarely engaged as citizens to consider the civic importance of immunization. As we have seen in the context of herd immunity, this skews the issues that are deemed important and the values that inform debates. However, the prominence of individual preferences in health contexts and society more generally, suggests civic discussion will require support to flourish. As Gutmann and Thompson observe, deliberation ‘will not suddenly turn self-centered individualists into public-spirited citizens…’ though they suggest it can help people take a broader view and ‘encourage public-spirited perspectives on public issues’ [63]. This does not acknowledge the extent to which parents enter an unfamiliar ethical landscape when they need to balance private and public interests. This makes it important for deliberation to include alternative ethical positions, an issue that is partially aided by our suggestion that relational autonomy should become standard in vaccination consultations. In addition, informing civic deliberation with principles at the heart of public health ethics - solidarity, public interest(s) and the use of proportionate constraints of individual autonomy [37] - would also help to counterbalance the prominence of individual, private concerns. Yet public health ethics has often focused on determining when limitations can be placed on individual freedoms, rather than on formulating a participatory approach to public health. In addition, it is not suitable for supporting clinical exchange on vaccination. Thus, efforts to address vaccine hesitancy must work to integrate different fields of ethics – clinical and public health ethics – within wider public debate on vaccination across health systems. Without such developments, future vaccination crises risk being fanned by suspicions created by conflicting ethical promises across clinical practice and health policy.

## Conclusion

Efforts to address vaccine hesitancy should avoid parallel, ethically conflicting strategies, or risk fueling suspicion in vaccination services. Rather than running independently, clinical and public health approaches need to be developed as an interdependent system. To do this effectively requires increasing the relational content of vaccination consultations and cultivating greater citizen deliberation on the tensions that hamper immunization uptake. Cultivating a more ethically consistent strategy will also require moving beyond the current silos of health ethics - clinical and public health ethics – to develop a more integrated approach for addressing vaccine hesitancy.

## Abbreviations

HCP: Healthcare professional
PCC: Parent-centered care
